# Allosteric Kinase Inhibitors Reshape MEK1 Kinase Activity Conformations in Cells and In Silico

**DOI:** 10.3390/biom11040518

**Published:** 2021-03-30

**Authors:** Jakob Fleischmann, Andreas Feichtner, Louis DeFalco, Valentina Kugler, Selina Schwaighofer, Roland G Huber, Eduard Stefan

**Affiliations:** 1 Institute of Biochemistry and Center for Molecular Biosciences Innsbruck, University of Innsbruck, Innrain 80/82, 6020 Innsbruck, Austria; jakob.fleischmann@uibk.ac.at (J.F.); andreas.feichtner@uibk.ac.at (A.F.); valentina.kugler@student.uibk.ac.at (V.K.); selina.schwaighofer@student.uibk.ac.at (S.S.); 2 Bioinformatics Institute (BII), Agency for Science Technology and Research (A*STAR), Singapore 138671, Singapore; louisd@bii.a-star.edu.sg (L.D.); rghuber@bii.a-star.edu.sg (R.G.H.); 3 Tyrolean Cancer Research Institute, Innrain 66, 6020 Innsbruck, Austria

**Keywords:** RAF, RAS, ERK, kinase inhibitor, allosteric inhibitor, cancer mutations, MAPK pathway, RAS–RAF–ERK pathway, personalized therapy, structure simulations, structure dynamics, biosensor, protein-fragment complementation assay, KinCon reporter

## Abstract

Mutations at different stages of the mitogen-activated protein kinase (MAPK) signaling pathway lead to aberrant activation of the involved protein kinase entities. These oncogenic modifications alter signal propagation which converge on the gatekeeper kinases MEK1/2, transmitting the input signal to ERK1/2. Thus, targeted MEK inhibition causes qualitative alterations of carcinogenic MAPK signals. Phosphorylation of the MEK1 activation loop at the positions S218 and S222 by RAF kinases triggers the conformational alignment of MEK’s catalytic pocket to enable ATP-binding and substrate phosphorylation. We have extended a kinase conformation (KinCon) biosensor platform to record MEK1 activity dynamics. In addition to MEK phosphorylation by BRAF, the integration of the phosphorylation-mimetic mutations S218D/S222D triggered opening of the kinase. Structural rearrangement may involve the flexibility of the N terminal MEK1 A-helix. Application of the allosterically acting MEK inhibitors (MEKi) trametinib, cobimentinib, refametinib, and selumetinib converted activated MEK1 KinCon reporters back into a more closed inactive conformation. We confirmed MEK1 KinCon activity dynamics upon drug engagement using the patient-derived melanoma cell line A2058, which harbors the V600E hotspot BRAF mutation. In order to confirm biosensor dynamics, we simulated structure dynamics of MEK1 kinase in the presence and absence of mutations and/or MEKi binding. We observed increased dynamics for the S218D/S222D double mutant particularly in the region of the distal A-helix and alpha-C helix. These data underline that MEK1 KinCon biosensors have the potential to be subjected to MEKi efficacy validations in an intact cell setting.

## 1. Introduction

Hyperactivation of components of the MAPK pathway contribute to the etiology and progression of cancer. At the plasma membrane, the RAS-GTPase family members HRAS, NRAS, and KRAS function as molecular switches for the activation of the downstream kinase cascade [1,2,3,4]. In human cancers, mutations of RAS variants, which occur primarily at three codon positions (G12, G13, Q61), cause constitutive RAS activation. Other signaling units of the MAPK pathway downstream of RAS show oncogenic mutations as well. Many mutations of BRAF have been reported, leading to constitutive kinase activation and thus decoupled phosphorylation events [5,6]. The most common gain of function BRAF mutation is the V600E substitution. This hotspot cancer mutation is found in around 60% of all malignant melanomas. BRAF-V600E circumvents the inhibitory effect of negative feedback regulation by ERK since it is not dependent on upstream regulation [4,7]. Uncoupled from RTK and RAS, BRAF-V600E is assumed to act as a functional monomer by constitutively activating the MEK kinases and thus cytoplasmic and nuclear ERK signaling [3,6,7]. Downstream of the RAF isoforms, MEK1 and MEK2 are activated as a result of RAF-mediated phosphorylation of MEK1 at the positions S218 and S222 of the kinase activation loop [8,9]. Both MEK1 and MEK2 are regarded to be the sole activators of ERK1 and ERK2 by phosphorylating evolutionary conserved residues [4,8]. MEK1/2 serve as critical gatekeeper kinases for numerous ERK functions. ERK kinases regulate various cellular events through the phosphorylation of more than 150 substrates in the cytoplasm and the nucleus, subsequently impacting cell proliferation and survival [8,10,11,12]. Unlike BRAF, activating mutations of MEK1 or MEK2 are found much less frequently in human tumors [13,14]. 

In recent years, the pharmaceutical targeting of the deregulated activities of the interconnected MAP-kinases BRAF, MEK, and ERK have been intensified. In a subset of tumors harboring kinase cancer driver mutations, the selective and direct blocking of mutant BRAF-dependent downstream signaling to MEK-ERK is a remarkable feature of BRAFi [5,15]. Tragically, kinase drug resistance occurs in the course of many cancer therapies [16,17,18]. These therapy drawbacks underline the necessity to develop other concepts which involve poly-pharmacological strategies. One resistance mechanism is linked to the dimerization of the inhibitor-bound BRAF protomer to a second wild-type RAF molecule. This leads to paradoxical RAF transactivation and thus to subsequent MEK activation and signal propagation to ERK [4,19]. This effect is most prominent in BRAF wild-type and/or RAS-mutated cell lines [20]. Thus, the combination therapy mixtures consisting of RAF and MEK specific blockers (e.g., trametinib (U.S. Food and Drug Administration (FDA)-approved MEKi) and dabrafenib (FDA-approved BRAFi)) have emerged as the standard therapy for melanoma patients harboring hotspot BRAF-V600E mutations [8,21]. Further, combination therapies using specific MEKi with other drugs are under investigation in current clinical trials [22]. MEK1/2 act as gatekeeper kinases of the MAPK pathway to nuclear targets. This central function of MEK kinases explains the rising interest in targeting the wildtype kinase functions in combinatorial cancer intervention approaches. However, kinase inhibitor therapies are confronted with the advent of acquired drug resistance. MEKi may lead to gain-of-function mutations or amplification of oncogenic genes which have to be considered for single and combination therapies and for the development of next-generation MEK inhibitors [6,8,23].

It is of interest that several MEKi such as trametinib, cobimentinib, refametinib, and selumetinib are not ATP-competitive and act allosterically [24]. They bind to a pocket distinct from the activation site, thus inducing a structural rearrangement which leads to the formation of a catalytically inactive kinase protein conformation [22,24,25]. Several kinases adopt different activity states which are reflected in specific alterations of their full-length protein conformations. Recently, we have developed a biosensor platform to record conformational dynamics of phosphotransferase activities in real time. This kinase conformation reporter system, which we have named KinCon, can be extended to a collection of full-length kinases for tracking their different kinase activity states [26]. In the proof-of-concept studies, we have shown that the engineered luciferase-based biosensors can be used to systematically track mutation and drug-driven changes of RAF kinase conformations in intact cell settings [26,27,28]. It is of interest that MEK kinases are smaller in size, when compared to RAF, but still they contain a N-terminal “negative regulatory region” which participates in stabilizing an inactive kinase conformation [8]. Recently we showed that very rare MEK1 patient mutations were sufficient to alter MEK1 kinase conformation states [27]. Here, we show that overexpression of active BRAF variants and the integrations of the phosphorylation-mimetic mutations S218D and S222D convert the KinCon biosensor to a more opened conformation. The MEKi trametinib, cobimentinib, refametinib, and selumetinib [24] promoted the structural rearrangement of the active MEK1 KinCon reporter. We have confirmed these cell-based observations using molecular dynamics simulations of apo and ligand-bound systems showing increased dynamics of the activated MEK1^S218D/S222D^ double-mutant kinase in particular. Thus, we propose that activated MEK1 KinCon biosensors have the potential to be used for efficacy validations of MEKi in different cell systems at expression levels below the endogenous kinase. 

## 2. Materials and Methods

### 2.1. Reagents

Benzyl-coelenterazine (Nanolight, Pinetop, Arizona, USA, #301,); Trametinib (Synonyms: GSK1120212; JTP-74057, MedChemExpress, Monmouth Junction, NJ, USA, HY10999), Cobimetinib (Synonyms: GDC-0973; XL518, MedChemExpress, HY13064), Refametinib (Synonyms: BAY 869766; RDEA119, MedChemExpress, HY-14691), Selumetinib (Synonyms: AZD6244; ARRY-142886, MedChemExpress, HY-50706), PLX8394 (MedChemExpress, HY18972).

### 2.2. Cell Culture and Antibodies

HEK293T (American Type Culture Collection (ATCC), Manassas, VA, USA; CRL-11268) and A2058 (ATCC CRL-11147) melanoma cells were grown in high glucose Dulbecco’s Modified Eagle’s Medium (DMEM) supplemented with 10% fetal bovine serum (FBS). Transient transfections of HEK293T cells were performed with Transfectin reagent (Biorad, Hercules, California, USA, #1703352). For transient overexpression of KinCon reporter constructs in the melanoma cell line A2058 (Figure 2E), 4 million cells were seeded on 10 cm dishes and cultivated overnight. Transfection was performed with jetPRIME transfection reagent (Polyplus-transfection, New York City, New York, USA, #114-07) according to the manufacturer’s instructions. Twenty-four hours post transfection, the cells were passaged and split on 96-well plates at a maximum density of 40,000 cells per well. At 48 h post transfection, drug exposure experiments and bioluminescence measurement were performed as described below. Primary antibodies used were the rabbit anti-GAPDH (Cell Signaling, Danvers, MA, USA, 2118S), rabbit anti-P-ERK1/2 (Cell Signaling, #9101), mouse anti-ERK1/2 (Cell Signaling #4696), rabbit anti-MEK1/2 and anti-P-MEK1/2 (Cell Signaling, #9126/#9154), mouse anti-FLAG^®^ M2-tag (Sigma-Aldrich, St. Louis, MO, USA, F3165-1MG).

### 2.3. Expression Constructs

KinCon reporter: Following PCR amplification of the human *MEK1* gene (MEK1: NM_002755.3), we fused it N-terminally with -F[1] and C-terminally with -F[2] of the *R*luc -PCA (pcDNA3.1 backbone vector) as previously described [27,28]. We inserted interjacent 10-aa standard linkers as previously described. A site-directed mutagenesis approach has been used to generate the MEK1 S218 and S222 amino acid substitutions to alanine, glutamic acid and aspartic acid, respectively. Flag-tagged MEK1: The Flag-tag was inserted C-terminally of MEK1 by PCR and cloned into the pcDNA3.1 vector using restriction enzymes.

### 2.4. Luciferase PCA Analyses

Indicated cells were grown in DMEM supplemented with 10% FBS. Indicated versions of the *R*luc PCA-based reporter were transiently overexpressed in 24-well plate formats (18 h expression, 130,000 cells seeded; 48 h expression, 90,000 cells seeded). Then, 18 or 48 h post-transfection, the drug exposure experiments were initiated. The growth medium was partially removed and kinase inhibitors were added to reach the final concentrations, as indicated in the figure legends. For the luciferase PCA measurements, the growth medium was carefully removed and the cells were washed with PBS. Cell suspensions were transferred to 96-well plates and subjected to luminescence analysis using the PHERAstar FSX (BMG labtech, Ortenberg, Germany). Luciferase luminescence signals were integrated for 10 s following addition of the *R*luc substrate benzyl-coelenterazine (Nanolight, #301).

### 2.5. Kinase Phosphorylation

Following overexpression of indicated flag-tagged MEK1 constructs in HEK293T cells, we directly determined the phosphorylation status of ERK1/2 via Western blotting with indicated antibodies. 48 h post transfection, we treated the cells with DMSO or with 10 nM, 100 nM, 500 nM, 1 µM or 10 µM of trametinib for 1 h. The medium was aspirated, cells were washed with cold 1× PBS and lysed in RIPA lysis buffer. Finally, 5× SDS loading buffer (LB) was added to reach a final concentration of 1× SDS LB.

### 2.6. Statistical Analyses

Data were examined for Gaussian distribution using the Kolmogorov–Smirnov normality test. Student’s *t*-tests were used to evaluate statistical significance. Values are expressed as the mean ± SEM as indicated. Significance was set at the 95% confidence level and ranked as * *p* < 0.05, ** *p* < 0.01, *** *p* < 0.001.

### 2.7. Molecular Dynamics Preparation

Apo MEK1 models were derived from PDB structures 1SJ9 and 3EQI, while ligand-bound systems were prepared using relevant crystal structures (4U7Z, 4LMN, and 3E8N for MEK1:G805, MEK1:GDC, and MEK1:RDEA respectively). Ligand parameters were described using the CGenFF server (version 3.0.1) and apo MEK1 and ligand-bound models were submitted to the H++ server (version 3.0) to determine protonation states of histidine residues at neutral pH [29,30]. MEK1 double mutants at S218 and S222 were mutated with PyMOL’s mutagenesis tool (Delano, W.L., 2002). All other ionizable residues were set to their default charged state. No cysteine residue is involved in disulfide bond formation. In all, thirteen unique systems were constructed: Apo WT MEK1, Apo, WT MEK1 2p (double phosphorylation at S218 and S222), Apo MEK1 S218D/E-S222D/E, G805 (selumetinib) WT MEK1, G805 MEK1 S218D/E-S222D/E, GDC0973 (cobimetinib) WT MEK1, GDC0973 MEK1 S218D/E-S222D/E, RDEA119 (refametinib) WT MEK1, RDEA119 MEK1 S128D/E-S222D/E. All systems are ATP-bound with coordinated Mg^2+^ ion constructed by superimposing ANP and the Mg^2+^ ion from PDB 4U7Z onto a given MEK1 model and converting the N3B nitrogen atom to oxygen. Protein interactions were modeled using the CHARMM36 force field [31]. All systems were solvated in a 0.15 M sodium chloride solution containing approximately 17,000 TIP3P water molecules. Solvation resulted in rectangular box sizes of approximately 8.2 × 8.2 × 8.2 nm.

### 2.8. Simulation Setup

All systems were equilibrated by performing 5000 steps of steepest descent minimization followed by 200 ps *NpT* ensemble simulations with gradually decreasing position restraints on the protein and ligand heavy atoms. All simulations were performed using GROMACS 2019.3. Electrostatic interactions were described using particle mesh Ewald [32]. Van-der-Waals and Ewald cut-offs were set to 1.1 nm. Bonds to hydrogen atoms were constrained with the LINCS algorithm, allowing an integration time step of 2 fs. Temperature was controlled for distinct coupling groups of solvent and solute using separate v-rescale thermostats [33] at 303.15 K, using a coupling constant τ of 1 ps. An isotropic Parrinello-Rahman barostat [34] maintained a pressure of 1 atm, using a coupling constant τ of 12 ps. Following equilibration, all systems were simulated for approximately 400 nanoseconds in the NpT ensemble. Frames were saved every 50 ps, yielding a single, continuous trajectory of at least 6800 frames for each system.

### 2.9. Simulation Analysis

All trajectories were aligned by their Cα atoms using *trjconv* from the GROMACS 2019.3 package with the fit flag and rot+trans option. Backbone root-mean-square deviation (RMSD) was then calculated using *rms* to determine the time point at which the system reaches equilibrium. Applying this time as a starting point, root mean square fluctuation (RMSF) analysis and angle measurements were performed on the aligned trajectory using *rmsf* and *angle*. Regions of particular interest comprise the activation segment, the conserved DFG motif containing D208, F209 and G210, [35] the αC helix, a hinge connecting N- and C-lobes, and the N-terminal A-helix. RMSF, average angle, and angle distributions of these regions as a function of simulation time were plotted for visual summarization. Three-dimensional representation of the Apo WT MEK1 model was prepared with the UCSF ChimeraX molecular visualization program [36].

## 3. Results

**Figure 1:**
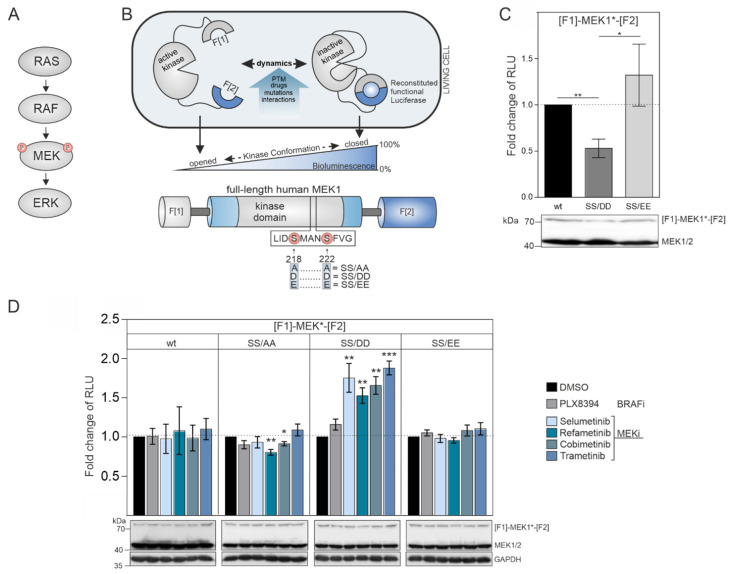
MEK1 KinCon reporter dynamics: (**A**) Simplified illustration of RAS-activated signal transmission within the RAS–RAF–MEK signaling pathway. (**B**) Schematic depiction of the MEK1 KinCon reporter principle indicating fragments 1 and 2 (= F[1]/F[2]) of the *R*luc-based PCA (top). Post-translational modifications (PTM), small-molecule binding, kinase domain mutations or protein–protein interactions may convert the MEK1 KinCon reporter into different kinase conformation states which are displayed by alterations of PCA-emitted bioluminescence signals. Domain organization of the MEK1 KinCon reporter iis shown. Activating phosphorylation sites are indicated. Their respective mutations to alanine (SS/AA), aspartic acid (SS/DD) and glutamic acid (SS/EE) are highlighted. (**C**) Effect of the phospho-mimetic site mutations S218D/S222D and S218E/S222E on MEK1 KinCon dynamics. Bars represent obtained bioluminescence signals in relative light units (RLU) relative to the signals of the wild-type MEK1 KinCon (± SEM from *n* = 6 independent experiments; normalized on KinCon reporter expression levels, (HEK293T)). (**D**) Dynamics of indicated MEK1 KinCon reporter signals upon exposure to four MEKi (1 µM), the RAFi PLX8394 (1 µM) or DMSO for 1 h. Bars represent the fold change of RLU relative to the DMSO control of the respective reporter entity (± SEM from at least *n* = 6 independent experiments, HEK293T). Student’s *t*-test was used to evaluate statistical significance. Confidence level is indicated by asterisk as: * *p* < 0.05, ** *p* < 0.01, *** *p* < 0.001.

The dual-specificity kinases MEK1/2 are the downstream targets of physiological and pathological RAF activities (Figure 1A) [8]. The phosphorylation of the MEK1 activation loop at the positions S218 and S222 by the upstream RAF kinases triggers the conformational alignment of the catalytic pocket to enable ATP and substrate binding. The consequence is the subsequent downstream phosphorylation of ERK1/2. We engineered a genetically encoded reporter for quantifications of intramolecular rearrangements of MEK1 kinase activity conformations. We set out to analyze the impact of phosphorylation-mimetic mutations in the activation loop, upstream RAF activities, and MEKi binding on MEK1 conformation states. We fused the coding region of full-length MEK1 N-terminally with fragment 1 (F[1]-) and C-terminally with fragment 2 (-F[2]) of the *Renilla* luciferase (*R*luc)-based protein-fragment complementation assay (PCA) to generate the reporter hybrid protein F[1]-MEK1-F[2] [37,38] which we termed MEK1 KinCon reporter (Figure 1B). MEK1 transmits input signals selectively to ERK1/2. The phosphorylation of MEK1 by RAF kinases at the positions S218 and S222 promotes MEK1 activation. First, we hypothesized that the unphosphorylated wild-type version of the MEK1 KinCon reporter should adopt a more closed inactive kinase conformation in the absence of upstream RAF kinase activities. Second, we assumed that MEK1 kinase activation could be achieved either upon BRAF-V600E co-expression or by mimicking RAF-induced phosphorylation at positions 218 and 222 through substitution of the Serine residues with negatively charged amino acids. These modifications may be sufficient to affect kinase structure and thus decrease the cellular KinCon bioluminescence signal (Figure 1B). To test our assumption, we performed transient expressions of wild-type and phosphorylation-mimetic MEK1 KinCon reporter constructs in HEK293T cells. We found that solely the S218D/S222D mutated MEK1 reporter yielded significantly decreased bioluminescence levels, while the glutamic acid KinCon mutant S218E/S222E showed no significant changes in bioluminescence values when compared with the wild-type KinCon reporter signals (Figure 1C).

These results indicate that the phospho-mimetic mutations S218D and S222D trigger ordering of the A-loop, disengagement of the N-terminal negative regulatory region and subsequent opening of MEK1 kinase conformation. This is in agreement with previous reports showing that aspartate mutants of MEK1 possess higher intrinsic in vitro kinase activity when compared to their glutamate counterparts [39,40,41]. Using the constructed collection of MEK1 KinCon reporters, we initiated KinCon inhibitor-profiling experiments. Following 48 h of KinCon overexpression in HEK293T cells, we specified expression levels of the biosensors to be far below the endogenous kinase. Next, we analyzed the impact of MEKi exposure (trametinib, refametinib, cobimetinib, selumetinib) on KinCon dynamics. All tested inhibitors promoted a selective transition of the S218D/S222D double-mutant KinCon from the opened conformation (exhibiting low bioluminescence levels) to a more closed kinase state (reflected by higher bioluminescence signals). The wild-type MEK1 KinCon reporter, the MEK1 KinCon^S218A/S222A^, and MEK1 KinCon^S218E/S222E^ showed no major reporter dynamics upon MEKi exposures for 1 h. The BRAFi PLX8394 acting as negative control had no effect on the tested MEK1 conformations (Figure 1D). 

**Figure 2:**
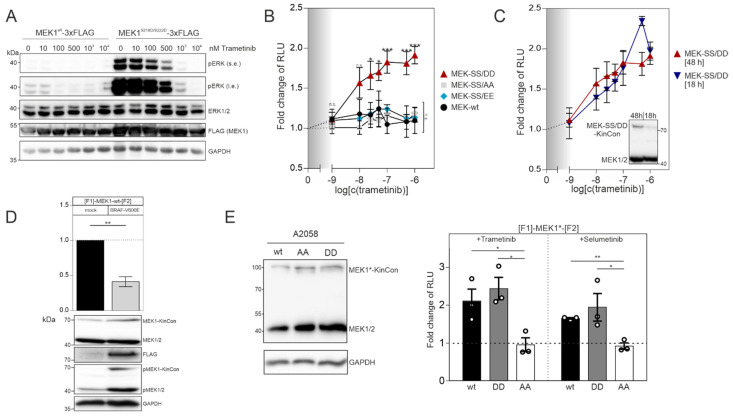
MEKi and BRAF activities affect MEK1 KinCon reporter dynamics: (**A**) ERK1/2 activation following overexpression of indicated MEK1 fusion proteins and exposure to increasing doses of the MEKi trametinib for 1 h. Shown is a representative Western blot of n = 4 independent experiments. Antibodies directed against phosphorylated ERK (pERK, either short exposure (s.e.) or long exposure (l.e.)), total ERK1/2, flag or GAPDH as loading control were used (HEK293T). (**B**) Dose-dependent effect of 1 h trametinib treatment on the dynamics of indicated MEK1 KinCon reporters. Data points represent obtained bioluminescence signals in RLU relative to the DMSO control. They were plotted against increasing inhibitor concentrations on a logarithmic scale (± SEM from *n* = 8 independent experiments, HEK293T). (**C**) Shown are the dose-dependent responses of trametinib exposure upon expression of the MEK1 KinCons for 18 h (*n* = 3) versus 48 h (*n* = 8) (HEK293T). (**D**) Bars represent obtained bioluminescence signals in RLU relative to the signals of the MEK1 KinCon reporter in the presence of overexpressed and flag-tagged BRAF-V600E (±SEM from *n* = 5 independent experiments; normalized on KinCon reporter expression levels). Antibodies directed against phosphorylated MEK1/2, total MEK1/2, flag and GAPDH were used (HEK293T). (**E**) A2058 transiently expressing indicated KinCons were subjected to MEKi exposure with 1 µM trametinib, selumetinib or DMSO for 1 h. Bioluminescence signals in relative light units (RLU) relative to the signals of the DMSO treatment are depicted (± SEM from *n* = 3 independent experiments). Student’s *t*-test was used to evaluate statistical significance. Confidence level is indicated by asterisk as: * *p* < 0.05, ** *p* < 0.01, *** *p* < 0.001.

In order to confirm that the MEK1 aspartate double mutant (S218D/S222D) represents the active kinase state, we transiently overexpressed the flag-tagged wild-type version and the S218D/S222D mutated MEK1 flag-fusion proteins in HEK293T cells. Following 48 h of expression, we detected that flag-tagged MEK1^S218D/S222D^ strongly elevated phospho-ERK1/2 levels, as predicted. In contrast to this, the phosphotransferase activity of overexpressed wild-type MEK1 was marginal (Figure 2A). Dose-dependent trametinib exposure reduced ERK1/2 phosphorylation and thus pathway activation, which is reflected by the decrease of ERK1/2 phosphorylation (Figure 2A). Next, we determined and compared the dose-dependent effect of the MEKi trametinib on indicated MEK1 KinCon reporter dynamics. We exposed HEK293T cells transiently expressing the aforementioned MEK1 KinCon reporters for 48 h to indicated doses of trametinib for 1 h. The wild-type, the double-alanine and the double-glutamate KinCon mutants were not affected by the treatment. In contrast, the double-aspartate mutant MEK1 KinCon showed a significant dose-dependent increase in the bioluminescence signal. This dynamic behavior underlines a progressive shift of the kinase conformation, upon MEKi treatment, towards the inactive kinase state (Figure 2B). Next, we decreased the expression of the MEK1^S218D/S222D^ KinCon by reducing the time of transient reporter expression to 18 h. The cells were subjected to the dose-dependent trametinib treatments as mentioned above. No significant differences between the dose–response curves for 48 h and 18 h of MEK1^S218D/S222D^ KinCon reporter expression were detectable (Figure 2C). 

MEK kinases are activated as a result of RAF-mediated phosphorylation of, e.g., MEK1 at the positions S218 and S222 of the kinase activation loop [8,9]. Thus, we set out to perform co-expression experiments of the catalytically active BRAF variant V600E and MEK1 KinCon. Following co-transfections of HEK293T cells with expression vectors for the wt MEK1 KinCon and flag-tagged BRAF-V600E in a ratio of 2:1, we observed an opening of the KinCon conformation. In line with this observation are the elevated phosphorylation levels of endogenous MEK1/2 and the transiently expressed MEK1-KinCon (Figure 2D). Next, we used the patient-derived melanoma cell line A2058 which harbors the catalytically active BRAF-V600E variant to validate the impact of indicated MEKi on reporter dynamics in a different cellular setting. Following overexpression of indicated KinCons for 48 h, we detected expression levels of the reporter fusion proteins far below the endogenous MEK1/2 kinases (Figure 2E, left). MEKi treatments (with the kinase blocker trametinib and selumetinib) of the patient-derived melanoma cells (purchased from ATCC) at a concentration of 1 µM for 1 h indeed triggered conformation dynamics. In contrast to the MEK1^S218A/S222A^ reporter, the wild-type and the activated MEK1^S218D/S222D^ KinCon reporters responded to drug exposure by promoting the more closed kinase conformation (Figure 2E, right). These data underline that the mutational customizing of MEK1 KinCon reporters provides a platform for tracking drug-driven changes of kinase activity conformations in the cell line of choice. 

The explicit inhibition of MEK activities causes qualitative alterations of MAPK signal propagation. The integration of the phospho-mimetic mutations S218D/S222D triggered the opening of the kinase conformation as we have tracked it in intact cells using KinCon reporter. Thus, we set out to validate our cellular MEK1 reporter measurements with simulations of structure dynamics in the presence and absence of MEKi and the respected MEK1 mutations of S218/S222. 

**Figure 3:**
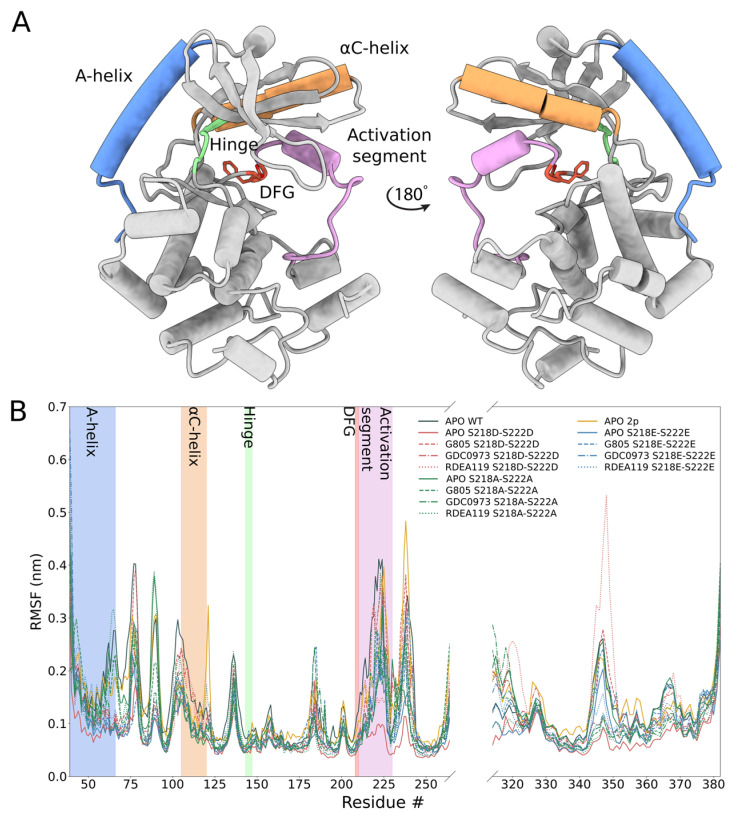
Structural overview of phosphorylation-mimetic MEK1 mutants: (**A**) Three-dimensional representation of the human MEK1 model used in MD simulation experiments. Coloring of secondary structure elements that undergo rearrangement during activation conforms with those regions highlighted in the RMSF analysis. Residue # represents amino acid position number. (**B**) Root-mean-square fluctuation (RMSF) analysis of WT MEK1, MEK1-S218D-S222D, MEK1-S218E-S222E, MEK1-S218A-S222A and corresponding ligand-bound complexes shows increased backbone displacement in and around regions engaged in kinase activation for Asp/Asp mutants relative to Glu/Glu and Ala/Ala mutants, signifying that open conformations are more prevalent in S218D-S222D systems.

**Figure 4:**
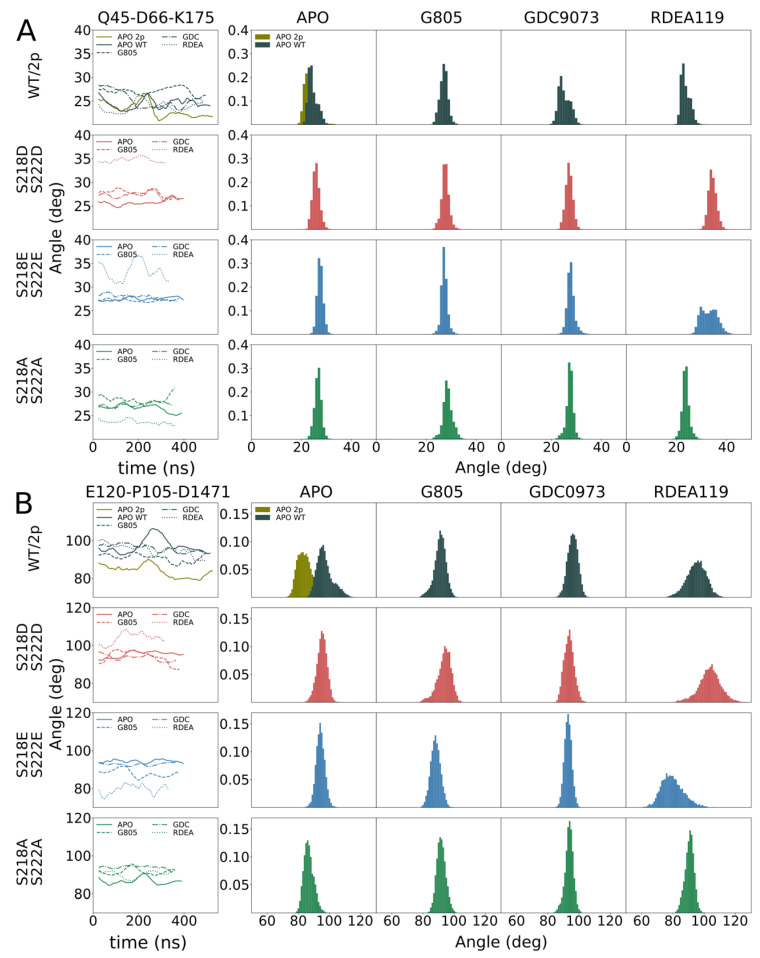
Structural dynamics of phosphorylation-mimetic MEK1 mutants: (**A**) MEK1 A-helix motion as characterized by a change in angle with respect to time where the observed angle is defined by Q45, D66, and K175 Cα atoms. The average angle over time for three systems (APO, G805 bound, and GDC0973 bound) suggests that Glu/Glu and Ala/Ala mutants adopt a narrower range of conformations compared to both Asp/Asp mutants and WT MEK1. (**B**) Similarly, MEK1 αC-helix motion is tracked by measuring the angle defined by αC-helix residues E120, P105, and hinge residue D147. Signature structural rearrangements that occur during kinase activation as expressed through αC-helix in/out conformations appear greatest between unphosphorylated and phosphorylated WT APO MEK1.

In order to identify the molecular origin of the observed differences, we conducted extensive molecular dynamics simulations of the WT, MEK1^S218D/S222D^, MEK1^S218E/S222E^, MEK1^S218A/S222A^ and dephosphorylated WT (Figure 3, Figure 4). The structure features of MEK1 are highlighted in Figure 3A. We observed that, in general, MEK1^S218D/S222D^ dynamics as measured by root mean square fluctuation (RMSF, Figure 3B) exceed dynamics observed for the MEK1^S218E/S222E^ variant. In particular, we investigated individual segments of MEK1 and identified the distal A-helix (Figure 3, Figure 4A) and the alpha-C helix (Figure 3, Figure 4B) as major regions of dynamic divergence. In general, MEK1^S218D/S222D^ exhibits significantly broader distributions of the angles describing their orientation relative to the kinase core. The variances of MEK1^S218D/S222D^ A-helix angles range from 2.1 to 2.6 degrees, whereas variances for MEK1^S218E/S222E^ are approximately half, at 1.2–1.6 degrees. Similarly, the variances of MEK1^S218D/S222D^ alpha-C helix angles range from 3.4 to 4.5 degrees, whereas variances for MEK1^S218E/S222E^ are considerably lower at 2.4–3.2 degrees, excepting RDEA119-bound complexes, which are highly variable across all systems. Such an increase in mobility is indicative of an enhanced propensity for structural rearrangement as indicated by the KinCon reporter system, and hence the findings are consistent with experimental evidence of structural change.

In summary, we successfully extended the KinCon biosensor concept to the dual-specificity kinase MEK1. The data obtained by the biosensor experiments suggest that BRAF-phosphorylated MEK1 along with the MEK1^-S218D/S222D^ KinCon reporters occupy an active and opened kinase conformation, which is in accordance with the shown structural MD simulation data. Treatment with allosteric MEKi exclusively affected the activated, phosphorylated and thus opened conformation states of the MEK1 wild-type reporter and the respective phospho-mimetic MEK1^-S218D/S222D^ mutant.

## 4. Discussion

Signaling units of the MAPK pathway are among the most frequently mutated oncogenic proteins. Therapeutic strategies to combat different types of cancer involve the inhibition of the participating kinases using defined RAFi and MEKi or their combinations [6,8,24,42]. In this study, we demonstrated the suitability of the cellular KinCon reporter system to track inhibitor binding. We apply customized KinCon reporters to record how allosterically acting MEKi interact with the catalytic cleft of activated MEK1 for reshaping its kinase conformation to a more inactive and closed full-length kinase state.

The cell-based demonstration of MEK1 kinase dynamics was affirmed in structural simulations, underlining that the S218D/S222D variant of MEK1 follows similar dynamics as the phosphorylated counterpart. Simulations of MEK1 WT, MEK1^S218D/S222D^, MEK1^S218E/S222E^ and MEK1^S218A/S222A^ revealed the molecular origin of the observed differential behavior. MEK1^S218D/S222D^ exhibited increased dynamics in both the A-helix and alpha-C helix compared particularly to the superficially analogous MEK1^S218E/S222E^ variant, as evidenced by a broadening in angle distributions under apo and ligand-bound conditions. Such broadening is consistent with behavior observed for the simulated di-phosphorylated system. Increased dynamics in the simulation showed that conformational stability for the MEK1^S218D/S222D^ variant is reduced, which resulted in an enhanced propensity for reporter activation. It would furthermore appear that ligand binding particularly affects MEK1^S218D/S222D^ in the region of the alpha-C helix with a more pronounced conformational shift observed for MEKi as compared to apo or any complex of MEK1^S218E/S222E^, which corresponded accurately with the reporter activation. Whereas our simulations show a consistent difference in behavior for the two phospho-mimetic mutations, we could not consistently observe a difference between WT and MEK1^S218D/S222D^. 

MEK1/2 relay physiological but also oncogenic input signals to ERK1/2. This function of MEK at a central crossroad of signal propagation has fostered drug discovery efforts to identify efficient MEKi. This led to the approval of a collection of non-ATP competitive MEKi. Amongst others, trametinib and cobimetinib found the way into the clinic for cancer therapy [8,24]. In contrast to ATP-competitive kinase inhibitors, these MEK1-specific bioactive small molecules do not need to compete with ATP [43]. Here, we show that these allosteric kinase inhibitors change the activity conformations selectively of the MEK1^S218D/S222D^ KinCon reporter. Moreover, we managed to track BRAF-V600E-mediated MEK1 KinCon activity states at expression levels of the KinCon well below the endogenous kinase. Thus, this approach seems to be suitable for kinase-targeted and cellular drug profiling efforts. This observation should be helpful for determining efficacies of drugs which interfere with the MAPK pathway using the MEK1 biosensor as read out in different cell settings. The complexity of kinase cascade regulations and the high incidence of cancer mutations require new means for recording cellular kinase rearrangements upon lead molecule binding to allosteric or ATP-competitive binding pockets. Such an approach might be suitable to track context-dependent kinase dynamics directly in the cell line of choice. The intact cell setting not only takes the whole spectrum of possible protein interactors into account, but also allows the imitation of the respective tumor microenvironment. Further, KinCon allows the implementation of patient mutations to assist in analyses of specific drug efficacies. The current focus of drug discovery and repurposing highly favors poly-pharmacological approaches. Here, KinCon offers high adaptability, practicability, and qualifies for high-content screening concepts for unveiling novel drug combinations.

## 5. Patents

Aspects of the present study are subject of pending patent applications.

## Data Availability

Data sharing not applicable.

## References

[1] Pylayeva-Gupta Y., Grabocka E., Bar-Sagi D. (2011). RAS oncogenes: Weaving a tumorigenic web. Nat. Rev. Cancer.

[2] Simanshu D.K., Nissley D.V., McCormick F. (2017). RAS Proteins and Their Regulators in Human Disease. Cell.

[3] Dorard C., Vucak G., Baccarini M. (2017). Deciphering the RAS/ERK pathway in vivo. Biochem. Soc. Trans..

[4] Lavoie H., Therrien M. (2015). Regulation of RAF protein kinases in ERK signalling. Nat. Rev. Mol. Cell Biol..

[5] Holderfield M., Deuker M.M., McCormick F., McMahon M. (2014). Targeting RAF kinases for cancer therapy: BRAF-mutated melanoma and beyond. Nat. Rev. Cancer.

[6] Yaeger R., Corcoran R.B. (2019). Targeting Alterations in the RAF-MEK Pathway. Cancer Discov..

[7] Karoulia Z., Gavathiotis E., Poulikakos P.I. (2017). New perspectives for targeting RAF kinase in human cancer. Nat. Rev. Cancer.

[8] Caunt C.J., Sale M.J., Smith P.D., Cook S.J. (2015). MEK1 and MEK2 inhibitors and cancer therapy: The long and winding road. Nat. Rev. Cancer.

[9] Roskoski R. (2012). MEK1/2 dual-specificity protein kinases: Structure and regulation. Biochem. Biophys. Res. Commun..

[10] Roskoski R. (2012). ERK1/2 MAP kinases: Structure, function, and regulation. Pharmacol. Res..

[11] Yoon S., Seger R. (2006). The extracellular signal-regulated kinase: Multiple substrates regulate diverse cellular functions. Growth Factors.

[12] Lake D., Correa S.A., Muller J. (2016). Negative feedback regulation of the ERK1/2 MAPK pathway. Cell Mol. Life Sci..

[13] Marks J.L., Gong Y., Chitale D., Golas B., McLellan M.D., Kasai Y., Ding L., Mardis E.R., Wilson R.K., Solit D. (2008). Novel MEK1 mutation identified by mutational analysis of epidermal growth factor receptor signaling pathway genes in lung adenocarcinoma. Cancer Res..

[14] Murugan A.K., Dong J., Xie J., Xing M. (2009). MEK1 mutations, but not ERK2 mutations, occur in melanomas and colon carcinomas, but none in thyroid carcinomas. Cell Cycle.

[15] Solit D.B., Rosen N. (2011). Resistance to BRAF inhibition in melanomas. N. Engl. J. Med..

[16] Lito P., Rosen N., Solit D.B. (2013). Tumor adaptation and resistance to RAF inhibitors. Nat. Med..

[17] Rauch N., Rukhlenko O.S., Kolch W., Kholodenko B.N. (2016). MAPK kinase signalling dynamics regulate cell fate decisions and drug resistance. Curr. Opin. Struct. Biol..

[18] Yao Z., Yaeger R., Rodrik-Outmezguine V.S., Tao A., Torres N.M., Chang M.T., Drosten M., Zhao H., Cecchi F., Hembrough T. (2017). Tumours with class 3 BRAF mutants are sensitive to the inhibition of activated RAS. Nature.

[19] Poulikakos P.I., Zhang C., Bollag G., Shokat K.M., Rosen N. (2010). RAF inhibitors transactivate RAF dimers and ERK signalling in cells with wild-type BRAF. Nature.

[20] Hatzivassiliou G., Song K., Yen I., Brandhuber B.J., Anderson D.J., Alvarado R., Ludlam M.J.C., Stokoe D., Gloor S.L., Vigers G. (2010). RAF inhibitors prime wild-type RAF to activate the MAPK pathway and enhance growth. Nature.

[21] Luke J.J., Flaherty K.T., Ribas A., Long G.V. (2017). Targeted agents and immunotherapies: Optimizing outcomes in melanoma. Nat. Rev. Clin. Oncol..

[22] Cheng Y., Tian H. (2017). Current Development Status of MEK Inhibitors. Molecules.

[23] Gao Y., Chang M.T., McKay D., Na N., Zhou B., Yaeger R.D., Torres N.M., Muniz K., Drosten M., Barbacid M. (2018). Allele-Specific Mechanisms of Activation of MEK1 Mutants Determine Their Properties. Cancer Discov..

[24] Wu P.K., Park J.I. (2015). MEK1/2 Inhibitors: Molecular Activity and Resistance Mechanisms. Semin. Oncol..

[25] Ohren J.F., Chen H., Pavlovsky A., Whitehead C., Zhang E., Kuffa P., Yan C., McConnell P., Spessard C., Banotai C. (2004). Structures of human MAP kinase kinase 1 (MEK1) and MEK2 describe novel noncompetitive kinase inhibition. Nat. Struct. Mol. Biol..

[26] Enzler F., Tschaikner P., Schneider R., Stefan E. (2020). KinCon: Cell-based recording of full-length kinase conformations. IUBMB Life.

[27] Mayrhofer J.E., Enzler F., Feichtner A., Röck R., Fleischmann J., Raffeiner A., Tschaikner P., Ogris E., Huber R.G., Hartl M. (2020). Mutation-oriented profiling of autoinhibitory kinase conformations predicts RAF inhibitor efficacies. Proc. Natl. Acad. Sci. USA.

[28] Rock R., Mayrhofer J.E., Torres-Quesada O., Enzler F., Raffeiner A., Raffeiner P., Feichtner A., Huber R.G., Koide S., Taylor S.S. (2019). BRAF inhibitors promote intermediate BRAF(V600E) conformations and binary interactions with activated RAS. Sci. Adv..

[29] Gordon J.C., Myers J.B., Folta T., Shoja V., Heath L.S., Onufriev A. (2005). H++: A server for estimating pKas and adding missing hydrogens to macromolecules. Nucleic Acids Res..

[30] Vanommeslaeghe K., Hatcher E., Acharya C., Kundu S., Zhong S., Shim J., Darian E., Guvench O., Lopes P., Vorobyov I. (2010). CHARMM general force field: A force field for drug-like molecules compatible with the CHARMM all-atom additive biological force fields. J. Comput. Chem..

[31] Huang J., MacKerell A.D. (2013). CHARMM36 all-atom additive protein force field: Validation based on comparison to NMR data. J. Comput. Chem..

[32] Darden T., York D., Pedersen L. (1993). Particle mesh Ewald: An N⋅log(N) method for Ewald sums in large systems. J. Chem. Phys..

[33] Bussi G., Donadio D., Parrinello M. (2007). Canonical sampling through velocity rescaling. J. Chem. Phys..

[34] Parrinello M., Rahman A. (1981). Polymorphic transitions in single crystals: A new molecular dynamics method. J. Appl. Phys..

[35] Kannan N., Neuwald A.F. (2005). Did protein kinase regulatory mechanisms evolve through elaboration of a simple structural component?. J. Mol. Biol..

[36] Goddard T.D., Huang C.C., Meng E.C., Pettersen E.F., Couch G.S., Morris J.H., Ferrin T.E. (2018). UCSF ChimeraX: Meeting modern challenges in visualization and analysis. Protein Sci..

[37] Röck R., Bachmann V., Bhang H.-E.C., Malleshaiah M., Raffeiner P., E Mayrhofer J., Tschaikner P.M., Bister K., Aanstad P., Pomper M.G. (2015). In-vivo detection of binary PKA network interactions upon activation of endogenous GPCRs. Sci. Rep..

[38] Bachmann V.A., Mayrhofer J.E., Ilouz R., Tschaikner P., Raffeiner P., Röck R., Courcelles M., Apelt F., Lu T.-W., Baillie G.S. (2016). Gpr161 anchoring of PKA consolidates GPCR and cAMP signaling. Proc. Natl. Acad. Sci. USA.

[39] Huang W., Erikson R.L. (1994). Constitutive activation of Mek1 by mutation of serine phosphorylation sites. Proc. Natl. Acad. Sci. USA.

[40] Mansour S.J., Candia J.M., Matsuura J.E., Manning M.C., Ahn N.G. (1996). Interdependent domains controlling the enzymatic activity of mitogen-activated protein kinase kinase 1. Biochemistry.

[41] Alessandrini A., Greulich H., Huang W., Erikson R.L. (1996). Mek1 phosphorylation site mutants activate Raf-1 in NIH 3T3 cells. J. Biol. Chem..

[42] Broman K.K., Dossett L.A., Sun J., Eroglu Z., Zager J.S. (2019). Update on BRAF and MEK inhibition for treatment of melanoma in metastatic, unresectable, and adjuvant settings. Expert Opin. Drug Saf..

[43] Roskoski R. (2017). Allosteric MEK1/2 inhibitors including cobimetanib and trametinib in the treatment of cutaneous melanomas. Pharmacol. Res..

